# Feasibility of Intraoperative Transoral Ultrasonography during the Sistrunk Procedure for Thyroglossal Duct Cysts Located on the Dorsal Side of the Hyoid Bone: A Case Report

**DOI:** 10.70352/scrj.cr.25-0496

**Published:** 2025-11-06

**Authors:** Masanaga Matsumoto, Yudai Goto, Akio Kawami, Hinako Sakai, Yuri Nemoto, Naoya Sakamoto, Kouji Masumoto

**Affiliations:** Department of Pediatric Surgery, Institute of Medicine, University of Tsukuba, Tsukuba, Ibaraki, Japan

**Keywords:** thyroglossal duct cysts, transoral ultrasonography, foramen cecum, recurrence, children

## Abstract

**INTRODUCTION:**

Thyroglossal duct cysts (TGDCs) are the most common congenital midline neck masses encountered in pediatric populations and are usually located anterior to the hyoid bone, making them readily identifiable by both superficial ultrasonography and skin palpation. However, intraoperative identification can be challenging in cases in which the cyst is located on the dorsal side of the hyoid bone or near the base of the tongue, which increases the risk of incomplete excision or rupture. This report describes the pediatric case of a TGDC located between the hyoid bone and the foramen cecum that was safely excised under intraoperative transoral ultrasonography (TOUS) guidance to facilitate identification of the entire cyst.

**CASE PRESENTATION:**

An 11-year-old boy was referred for evaluation of an incidentally detected midline neck mass. Neck ultrasonography and MRI revealed the presence of a 7-mm cyst located between the hyoid bone and the foramen cecum, consistent with the characteristics of a TGDC, and a Sistrunk procedure was scheduled. Intraoperatively, the cyst was successfully identified using TOUS with a small convex probe, which provided a stable and continuous view from the oral side throughout the dissection. A transverse cervical incision was made, the central hyoid bone was removed, and the cyst was visualized on its dorsal side under TOUS guidance. En bloc resection of the entire cyst and tract was completed without rupture, and histopathology confirmed the diagnosis of TGDC. The postoperative course was uneventful, and no recurrence was observed at the 4-month follow-up assessment.

**CONCLUSIONS:**

The use of TOUS enabled real-time visualization of a deep TGDC structure that was difficult to identify via superficial ultrasonography after neck incision. Thus, TOUS can serve as a reliable guide during the Sistrunk procedure, reducing the risk of cyst rupture and incomplete resection, thereby enabling safe and complete excision. The experience of this case highlights the potential benefit of using TOUS in managing deep TGDCs located on the dorsal side of the hyoid bone, especially in pediatric patients.

## Abbreviations

TGDCs
: thyroglossal duct cysts
TOUS
: transoral ultrasonography

## INTRODUCTION

TGDCs are the most common type of congenital anomaly of the neck in pediatric populations,^1)^ accounting for more than 70% of all congenital cervical anomaly cases.^2)^ TGDCs are typically located in the midline and anterior portion of the neck,^1,3)^ and superficial ultrasonography is frequently employed for their diagnosis owing to the accessibility and noninvasive nature of the technique.^2,4)^ The standard treatment for TGDCs is the Sistrunk procedure. The reported recurrence rates following surgery have been shown to range from 0% to 12.2%,^5)^ and the primary cause of recurrence is the incomplete removal of the cyst or tract.^1,6)^

Deep cysts, such as those located on the dorsal side of the hyoid bone or near the base of the tongue, may be associated with an elevated risk of recurrence following incomplete excision or intraoperative rupture. Caniglia et al.^7)^ reported that TGDCs located on the dorsal side of the hyoid bone were prone to recurrence owing to the increased difficulty of surgical removal. In such cases, the use of intraoperative real-time imaging to localize deep cysts could reduce the risk of incomplete resection and recurrence.

This report describes a pediatric case involving a TGDC located between the hyoid bone and the foramen cecum that was safely excised under intraoperative TOUS guidance to facilitate the identification of the entire cyst.

## CASE PRESENTATION

**Fig. 1:**
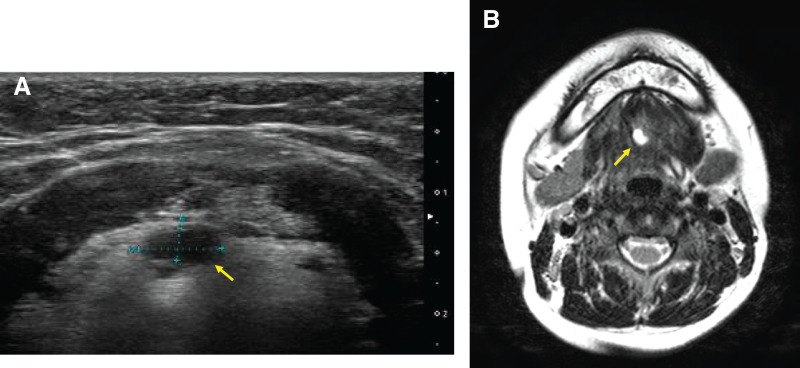
Preoperative findings. (**A**) Transcervical ultrasonography reveals a 7-mm cystic lesion (arrow) located on the dorsal side of the hyoid bone. (**B**) Neck MRI confirms the presence of a simple cyst (10 × 7 × 3 mm in size, arrow) near the midline between the hyoid bone and the foramen cecum.

An 11-year-old boy was referred for evaluation of a midline neck cyst that was incidentally detected via neck ultrasonography during assessment for diffuse thyroid enlargement. He had no symptoms related to swallowing or breathing difficulties, and he had not experienced any vocal changes. Physical examination revealed symmetric enlargement of the thyroid gland. There were no sinus tract openings or overlying skin changes. There was no history of neck infection. Laboratory evaluations revealed positive anti-thyroid peroxidase antibody labeling, consistent with suspected early-stage Hashimoto thyroiditis, and he was scheduled for conservative follow-up under the care of a pediatrician. Neck ultrasonography and subsequent MRI revealed a cystic lesion located between the hyoid bone and the foramen cecum (**Fig. 1A**, **1B**), the characteristics of which were consistent with a TGDC. Based on these findings, a Sistrunk procedure was scheduled.

**Fig. 2:**
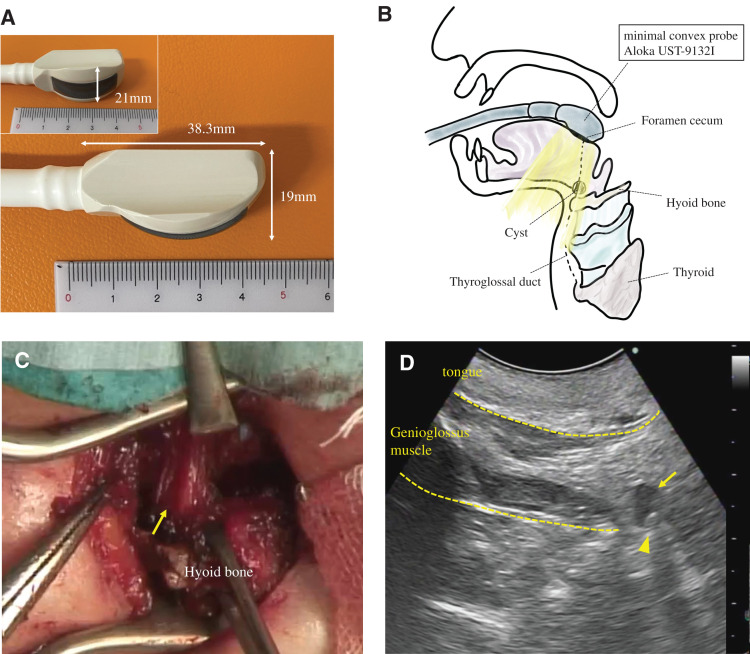
Intraoperative transoral ultrasonography (TOUS) as a guide for cyst identification. (**A**) Photograph of the minimal convex probe (Aloka UST-9132I Intraoperative Convex, ProSound Finger-grip; HITACHI Healthcare Manufacturing, Kashiwa, Japan). The probe head measures 38.3 × 19 × 21 mm. (**B**) Schematic illustration showing transoral insertion of the minimal convex probe, with the tip positioned near the foramen cecum in the midline at the tongue base. Intraoperative TOUS can delineate the cyst located on the dorsal and cranial side of the hyoid bone. (**C**) Intraoperative view following resection of the central portion of the hyoid bone. A muscle cuff (arrow) is observed on the dorsal and cranial side of the hyoid bone, representing the presumed site of the thyroglossal duct cyst and tract. (**D**) Intraoperative TOUS images of the tongue and the underlying genioglossus muscle. The cystic lesion (arrow) is identified within the muscle cuff, the accurate position of which was further verified by gently probing the area with forceps arrowhead).

**Fig. 3:**
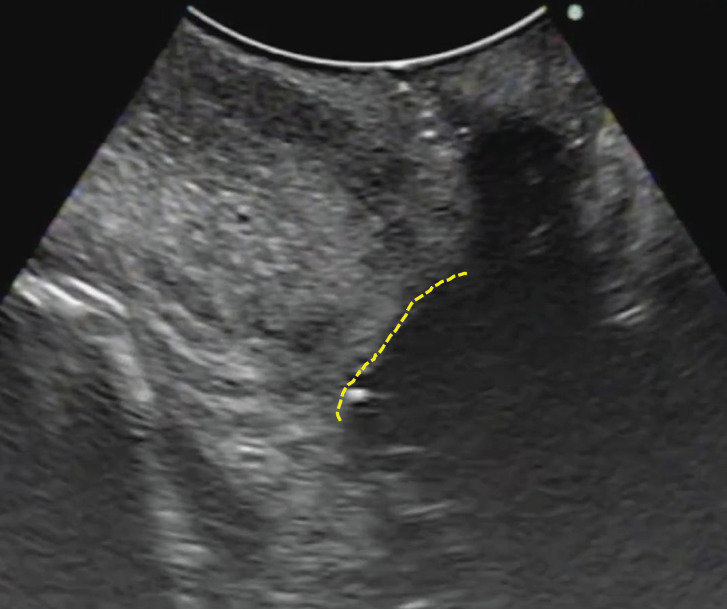
Intraoperative transoral ultrasonography (TOUS) image following en bloc resection TOUS image following en bloc resection of the cyst, tract, and central portion of the hyoid bone. The dotted line indicates the tongue-side dissection plane. No residual cystic structure is observed, confirming complete excision.

Under general anesthesia, the patient was placed in the supine position to allow for neck extension, endotracheal intubation was performed, and the tube was placed on the right side of the oral cavity. TOUS was employed using a minimal convex probe (Aloka UST-9132I Intraoperative Convex – ProSound Finger-grip, HITACHI Healthcare Manufacturing, Kashiwa, Japan), with a probe head measuring 38.3 × 19 × 21 mm, to identify and localize the cyst behind the hyoid bone (**Fig. 2A**, **2B**). The probe was inserted midline into the oral cavity before the start of surgery and gently pressed against the tongue-side oral mucosa as needed, allowing continuous and clear visualization of the cyst. A 2.5-cm transverse cervical incision was made, approximately 20 mm of the central hyoid bone was excised, and the cyst was identified on the dorsal aspect of the hyoid bone under TOUS guidance (**Fig. 2C**, **2D**). Using TOUS, the entire cyst was delineated, the cranial portion of the tract was ligated near the foramen cecum, and the cyst, tract, and hyoid bone were removed en bloc without rupture (**Fig. 3**).

**Fig. 4:**
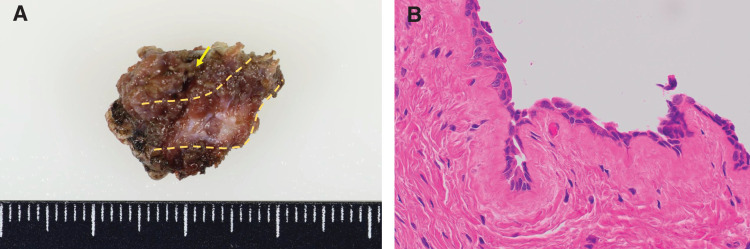
Gross and microscopic pathologic findings of the excised thyroglossal duct cyst and hyoid bone. (**A**) Gross specimen shows the excised central portion of the hyoid bone (dotted line) and the cyst (arrow) located on the dorsal side of it. (**B**) Microscopic finding demonstrates the epithelial lining of the cyst wall, consistent with a thyroglossal duct remnant (hematoxylin and eosin staining, magnification ×40).

Histopathological examination confirmed the diagnosis of a TGDC, which was lined with pseudostratified columnar epithelium, and there were no signs of malignancy (**Fig. 4**). The postoperative course was uneventful, and the patient was discharged on POD 2. At the 4-month follow-up assessment, there was no evidence of recurrence.

## DISCUSSION

In this case, intraoperative TOUS facilitated the safe excision of a deep TGDC located between the hyoid bone and the foramen cecum without rupture. The imaging technique provided a fixed view from the foramen cecum side throughout the surgical procedure, serving as an accurate guide. The cyst remained visible in the same field, even during deeper dissection, and its position could be confirmed directly using surgical forceps under ultrasonographic guidance. This facilitated the precise localization and delineation of the dissection line while referencing the relationship between the cyst and the foramen cecum.

In adult patients, TOUS has been reported to be a useful tool, not only for the diagnosis of head and neck masses but also as an intraoperative guide during biopsy and surgical procedures.^8–12)^ Furthermore, intraoperative TOUS has been shown to be useful in assessing the depth of invasion in cases of tongue cancer^9)^ and in improving the detection and visualization of oropharyngeal tumors,^10)^ thus making it a valuable alternative to conventional transcervical ultrasonography. Vu et al.^11)^ reported several advantages of TOUS, including the ability to evaluate retropharyngeal lesions in both the transverse and sagittal planes, whereas the use of cervical ultrasonography is limited in this region owing to the presence of overlying osseous structures. Additionally, Fornage et al.^12)^ reported that in both fine-needle aspiration biopsy and resection of retropharyngeal masses, the use of TOUS with real-time and color Doppler capabilities can facilitate assessments of the presence or absence of a fat plane between the lesion and the carotid artery, which is critical information for surgeons to consider when performing such procedures. The successful use of TOUS according to such reports prompted us to assess whether TOUS could be applied in surgical procedures to treat deep TGDCs in a pediatric patient using a small convex probe. Some small convex probes are capable of both sagittal and transverse imaging, potentially allowing for better visualization in cases involving the lateral deviation of lesions, and TOUS was technically feasible, even in a school-aged child as was the case with our patient.

During the Sistrunk procedure, surgeons have traditionally inserted a finger into the oral cavity to estimate the direction and depth of the thyroglossal tract toward the foramen cecum.^13)^ TOUS can serve a similar role when the probe is applied against the oral mucosa and gently pressed toward the foramen cecum, allowing physical manipulation of the surgical field and visual navigation. This method allows for real-time visual navigation that can be shared with all members of the surgical team, potentially improving both the safety and precision of the surgical procedures being performed.

Although TOUS was feasible in our school-aged patient, its application in younger children under general anesthesia with transoral intubation may be limited by the smaller oral cavity, as the probe may not adequately reach the tongue base. Furthermore, unlike in adults, TOUS may be difficult to use for diagnostic purposes in children in the outpatient setting. Therefore, the intraoperative TOUS view is typically obtained for the 1st time during surgery. It is thus important to anticipate the intraoperative sonographic anatomy in advance by reviewing preoperative images such as sagittal MRI.

## CONCLUSIONS

TOUS can serve as a feasible and helpful intraoperative tool to assist in the safe excision of deep TGDCs located between the hyoid bone and the foramen cecum, even in pediatric patients.

## DECLARATIONS

### Availability of data and materials

Data sharing is not applicable to this article as no datasets were generated or analyzed during the current study.

## References

[1] Mondin V, Ferlito A, Muzzi E, et al. Thyroglossal duct cyst: Personal experience and literature review. Auris Nasus Larynx 2008; 35: 11–25. 17720342 10.1016/j.anl.2007.06.001

[2] Lin ST, Tseng FY, Hsu CJ, et al. Thyroglossal duct cyst: a comparison between children and adults. Am J Otolaryngol 2008; 29: 83–7. 18314017 10.1016/j.amjoto.2007.02.003

[3] Maddalozzo J, Alderfer J, Modi V. Posterior hyoid space as related to excision of the thyroglossal duct cyst. Laryngoscope 2010; 120: 1773–8. 20715087 10.1002/lary.21043

[4] Chou J, Walters A, Hage R, et al. Thyroglossal duct cysts: anatomy, embryology and treatment. Surg Radiol Anat 2013; 35: 875–81. 23689821 10.1007/s00276-013-1115-3

[5] Sistrunk WE. The surgical treatment of cysts of the thyroglossal tract. Ann Surg 1920; 71: 121–2. 17864229 10.1097/00000658-192002000-00002 PMC1410396

[6] Ducic Y, Chou S, Drkulec J, et al. Recurrent thyroglossal duct cysts: a clinical and pathologic analysis. Int J Pediatr Otorhinolaryngol 1998; 44: 47–50. 9720680 10.1016/s0165-5876(98)00041-x

[7] Caniglia AJ, Johnston DR, Rastatter JC, et al. Knowledge and utilization of the posterior hyoid space as related to excision of the thyroglossal duct cyst. Laryngoscope 2022; 132: 668–9. 34581448 10.1002/lary.29840

[8] Clayburgh DR, Byrd JK, Bonfili J, et al. Intraoperative ultrasonography during transoral robotic surgery. Ann Otol Rhinol Laryngol 2016; 125: 37–42. 26215725 10.1177/0003489415596754 PMC5458621

[9] Kaltoft M, Hahn CH, Wessman M, et al. Intraoral ultrasound versus MRI for depth of invasion measurement in oral tongue squamous cell carcinoma: a prospective diagnostic accuracy study. Cancers (Basel) 2024; 16: 637. 38339388 10.3390/cancers16030637 PMC10854529

[10] Garset-Zamani M, Norling R, Hahn CH, et al. Transoral ultrasound in the outpatient clinic for the diagnostic work-up of oropharyngeal cancer: a feasibility study. Cancers (Basel) 2023; 15: 5292. 37958465 10.3390/cancers15215292 PMC10649062

[11] Vu TH, Kwon M, Ahmed S, et al. Diagnostic accuracy and scope of intraoperative transoral ultrasound and transoral ultrasound-guided fine-needle aspiration of retropharyngeal masses. AJNR Am J Neuroradiol 2019; 40: 1960–4. 31582388 10.3174/ajnr.A6236 PMC6975117

[12] Fornage BD, Edeiken BS, Clayman GL. Use of transoral sonography with an endocavitary transducer in diagnosis, fine-needle aspiration biopsy, and intraoperative localization of retropharyngeal masses. AJR Am J Roentgenol 2014; 202: W481–6. 24758683 10.2214/AJR.13.11398

[13] Marshall SF, Becker WF. Thyroglossal cysts and sinuses. Ann Surg 1949; 129: 642–51. 17859346 PMC1514138

